# Homozygosity for the SCN10A Polymorphism rs6795970 Is Associated With Hypoalgesic Inflammatory Bowel Disease Phenotype

**DOI:** 10.3389/fmed.2018.00324

**Published:** 2018-11-27

**Authors:** Eugene Gonzalez-Lopez, Yuka Imamura Kawasawa, Vonn Walter, Lijun Zhang, Walter A. Koltun, Xuemei Huang, Kent E. Vrana, Matthew D. Coates

**Affiliations:** ^1^Department of Pharmacology, Penn State College of Medicine, Hershey, PA, United States; ^2^Departments of Pharmacology and Biochemistry & Molecular Biology, Institute for Personalized Medicine, Penn State College of Medicine, Hershey, PA, United States; ^3^Department of Public Health Sciences, Penn State College of Medicine, Hershey, PA, United States; ^4^Department of Biochemistry and Molecular Biology, Penn State College of Medicine, Hershey, PA, United States; ^5^Division of Colorectal Surgery, Department of Surgery, Penn State College of Medicine, Hershey, PA, United States; ^6^Department of Neurology, Penn State College of Medicine, Hershey, PA, United States; ^7^Division of Gastroenterology & Hepatology, Department of Medicine, Penn State College of Medicine, Hershey, PA, United States

**Keywords:** genetic polymorphism, inflammatory bowel disease, voltage-gated sodium channels, Na_v_1.8, SCN10A, hypoalgesia, abdominal pain

## Abstract

**Background:** Hypoalgesic inflammatory bowel disease (IBD), a condition in which patients with active disease do not perceive and/or report abdominal pain, is associated with serious complications and there is a lack of cost-effective, reliable diagnostic methods to identify “at-risk” patients. The voltage-gated sodium channels (VGSC's), Na_v_1.7, Na_v_1.8, and Na_v_1.9, are preferentially expressed on nociceptive neurons, and have been implicated in visceral inflammatory pain. At least 29 VGSC single nucleotide polymorphisms (SNPs) have been implicated in chronic somatic pain syndromes, but little is known about their role in human visceral sensation. We hypothesized that disruptive VGSC polymorphisms result in anti-nociceptive behavior in IBD.

**Methods and Findings:** We performed targeted exome sequencing and/or TaqMan genotyping to evaluate the Na_v_1.7, Na_v_1.8, and Na_v_1.9 genes (SCN9A, SCN10A and SCN11A) in 121 IBD patients (including 41 “hypoalgesic” IBD patients) and 86 healthy controls. Allelic and genotypic frequencies of polymorphisms were compared among study groups who had undergone characterization of intestinal inflammatory status and abdominal pain experience. Forty-nine total exonic SNPs were identified. The allelic frequency of only one non-synonymous SNP (rs6795970 [*SCN10A*]) approached significance in hypoalgesic IBD patients when compared to other IBD patients (*p* = 0.096, Fisher's exact test). Hypoalgesic IBD patients were more likely to be homozygous for this polymorphism (46 vs. 22%, *p* = 0.01, Fisher's exact test).

**Conclusions:** This is the first human study to demonstrate a link between a genetic variant of SCN10A and abdominal pain perception in IBD. These findings provide key insights into visceral nociceptive physiology and new diagnostic and therapeutic targets to consider in IBD and other gastrointestinal conditions associated with chronic abdominal pain. Further studies are required to elucidate the precise pathophysiological impact of the rs6795970 polymorphism on human gastrointestinal nociception.

## Introduction

Inflammatory Bowel Disease (IBD) encompasses a group of disorders, including Crohn's disease (CD) and ulcerative colitis (UC), characterized by chronic relapsing inflammation of the gastrointestinal (GI) tract. IBD affects as many as 3 million Americans, most of whom are diagnosed before age 35 (1). These disorders are chronic, life-long conditions that can be treated, but currently do not have a cure. Chronic abdominal pain is one of the major reasons individuals with IBD seek medical attention (2) and is described in up to 70% of patients at the onset or during exacerbations of the disease (3). The inflammation associated with IBD is considered to be a primary driver of this pain, as pro-inflammatory mediators sensitize extrinsic sensory neurons that project to the gut.

In some instances, however, the lack of abdominal pain in IBD can also pose different but significant challenges. Individuals with so-called hypoalgesic and/or “silent” IBD have grossly evident intestinal inflammatory changes that do not produce significantly painful or other noxious sensations. It has been increasingly recognized that symptom-based assessment tools can be relatively insensitive for accurately assessing IBD activity (4). Various estimates have suggested that a third or more of IBD patients with active disease will be asymptomatic (5, 6). This is important considering that individuals with “silent” IBD are less likely to seek appropriate medical attention and more likely to develop complications (including strictures, fistulae, and abscesses) and ultimately incur major healthcare costs, including hospitalization (6).

Voltage-gated sodium channels (VGSC's) appear to play an important role in visceral pain perception (7). In particular, three VGSC's (Na_v_1.7, Na_v_1.8, and Na_v_1.9) have recently been implicated as primary mediators of visceral nociceptive function, including that associated with the gastrointestinal tract. Investigations incorporating retrograde labeling techniques have demonstrated the presence of these channels in nociceptive neurons innervating the stomach, small intestine, and colon (7–12). The electrophysiological profile of these cells suggests that Na_v_1.8 is particularly important for their normal function (12–14). In support of this idea, studies assessing nociceptive response in animal models utilizing chemically induced colitis successfully attenuate the associated pain using either therapeutics (15) or genetic knock-outs that target Na_v_1.8 (16). We hypothesized that genetic variants inducing a “loss-of-function” in one or more of the genes associated with Na_V_1.7, Na_V_1.8, or Na_V_1.9 (SCN9A, SCN10A, and SCN11A, respectively) in IBD patients would result in anti-nociceptive function and diminished abdominal pain perception.

## Materials and methods

### Study participant selection

We obtained relevant clinical and patient survey data from individuals who had consented to take part in a prospective IBD natural history registry and tissue biorepository associated with the IBD Center at Penn State Hershey Medical Center [approved by the Penn State College of Medicine Institutional Review Board (PRAMSHY98-057)] and who had undergone a colonoscopy between October 1, 2015 and January 31, 2017.

#### Inclusion criteria

We included patients with established diagnoses of IBD who had (1) undergone a colonoscopy, (2) completed a pain questionnaire (see below) at the time of endoscopy, and (3) had provided a blood sample as part of their participation in the registry and biorepository. In addition, IBD study participants had to meet the following criteria: (1) age ≥ 18 years; (2) established diagnosis of IBD (either Crohn's Disease or Ulcerative Colitis, based upon standard clinical criteria incorporating historical, laboratory, endoscopic, and histological evaluation); (3) no coexisting condition that could explain abdominal pain, including pregnancy, trauma, malignancy, infection, or non-IBD associated inflammatory disorder. We also included a cohort of healthy adult (age ≥ 18 years) control patients who had no documented history of chronic gastrointestinal illness or known pain disorder.

#### Exclusion criteria

IBD patients were excluded if they had an indeterminate form of IBD, microscopic colitis, inflammatory enteritis or colitis not associated with IBD or had not provided information about pain at or within 1 month of the colonoscopy. Patients were also excluded if they had undergone a total colectomy or proctocolectomy at any time or any intra-abdominal surgery within the calendar year prior to the time of the study encounter. Healthy controls were excluded if they had a history of chronic gastrointestinal disease or acute or chronic abdominal pain or other pain disorder.

### Characterization of intestinal inflammatory status and complications

Disease severity and location were recorded for each IBD study participant utilizing contemporary endoscopic, histologic, and radiologic information. CD and UC location and phenotype were classified according to the Montreal Classification system (17). The severity of UC disease activity was determined based upon the appearance of the mucosa at the time of endoscopy and characterized using the Mayo Clinic endoscopy sub-score (18). The severity of CD was also determined by appearance at endoscopy using the Crohn's Disease Simple Endoscopic Score (SES-CD) (18). Both the Mayo score and the SES-CD are based upon a Likert-type scale ranging from 0 to 3 (with 0 = inactive, 1 = mild activity, 2 = moderate activity and 3 = severe activity). All complications described were intra-abdominal/luminal in nature (e.g., abscesses and fistulae described were not perianal phenomena).

### Abdominal pain assessment

Abdominal pain ratings were based primarily on responses to the Short Inflammatory Bowel Disease Questionnaire (SIBDQ), that asks patients to grade pain on a frequency-based inverse Likert scale (“How often over the past 2 weeks have you been troubled by pain in the abdomen?”), with 1 representing pain “all of the time” and 7 representing pain “none of the time” (19). We also obtained information about pain severity using responses to an ulcerative colitis activity index survey, that asks about the severity of abdominal pain (with potential responses including 0 (“no abdominal pain”), 1 (“mild”), 2 (“moderate”) and 3 (“severe”). As abdominal pain frequency and intensity scores correlated with one another (*r* = −0.7, *p* < 0.001), we operationally defined presence of clinically meaningful abdominal pain as SIBDQ pain ratings of ≤ 5 (with 5 defined as “a little of the time”).

### Determination of study cohorts

There were three cohorts evaluated during this study: (1) IBD patients with active disease who had an SIBDQ pain score of >5 (describing “hardly any” to “no” abdominal pain) (i.e., active disease with no pain (ANP) or “hypoalgesic IBD”), (2) all other IBD patients, and (3) healthy control patients. Basic demographic and disease characteristics of each group can be found in Table 1. Of note, all patients identified as having active disease had a Mayo or SES-CD score of 2 or greater. As described above, all patients with an SIBDQ pain score of ≤ 5 were defined as having abdominal pain.

**Table 1:** Study participant demographic and disease characteristics.

	**“Hypoalgesic IBD” (*****n*** = **41)**		**Other IBD (*****n*** = **80)**		**Controls (*n* = 86)**	***p*****-value**	
Age (years)	41.8 ± 2.6		41.7 ± 1.5		63.3 ± 1.1	< 0.0001	
Gender (female:male)	15:26		43:37		42:44	0.20	
Disease type (CD:UC)	25:16		52:28		–	0.69	
Disease location	CD	UC	CD	UC		CD	UC
(Montreal classification)	L1: 4	E1: 0	L1: 7	E1: 0		0.74	1
	L2: 5	E2: 4	L2: 14	E2: 10		0.58	0.52
	L3: 16	E3: 12	L3: 31	E3: 18		0.99	0.52
	L4: 0		L4: 0			1
Disease duration (years)	11.5 ± 1.3		13.4 ± 1.0		–	0.28	
History of stricture	15		8		–	0.001	
History of fistula	8		3		–	0.007	
History of abscess	9		3		–	0.003	
History of colon cancer	1		0		–	0.34	

*Using the Montreal Classification system for localizing disease activity, for Crohn's disease, L1, terminal ileum; L2, colon; L3, ileocolonic and L4, upper gastrointestinal tract; while for ulcerative colitis; E1, proctitis; E2, left sided colitis, and E3, pan-colitis. IBD, inflammatory bowel disease; CD, Crohn's Disease; UC, ulcerative colitis*.

### DNA isolation

High-quality genomic DNA was isolated from whole blood using silica-based spin columns (QIAmp DNeasy Blood & Tissue Kit, Qiagen, Hilden, Germany). Spectrophotometry was used to quantify DNA and the quality of the isolated material was evaluated with an Agilent Bioanalyzer.

### Custom target capture sequencing and analysis

The custom capture oligo set was designed by the SureDesign platform from Agilent Technologies (SureSelectXT Custom Capture Oligo) against *SCN9A, SCN10A*, and *SCN11A*. This set was specifically designed to capture all exons, the proximal promoter sequence, as well as limited intron sequences and untranslated regions near exons. The target-captured sequencing libraries were constructed using the KAPA LTP Library Preparation Kit (Kapa Biosystems, Inc., Wilmington, MA) combined with a SureSelectXT Reagent Kit (Agilent Technologies), and sequenced on an Illumina Hiseq 2500 sequencer (Illumina, Inc, San Diego, CA) at read length of paired-end 2 × 100 bp. Targeted exome sequencing was performed on an initial cohort of 46 IBD patients that included a mixture of individuals with and without inflammation and abdominal pain. Generated reads were aligned with the GRCh37 human reference genome using the Burrows-Wheeler alignment (20). Variant detection and analysis were performed using the GATK Best Practice for germline SNP/indel finding workflow (Broad Institute). ANNOVAR software (21) was used to annotate the variants and identify synonymous, non-synonymous, and deleterious variants for further analysis.

### TaqMan SNP genotyping

For rigor and reproducibility, genotype analysis of all 46 IBD samples described above, along with samples from an additional 75 IBD patients (121 total) and 86 healthy control patients, was performed with commercially available TaqMan assays using the OpenArray platform on a QuantStudio 12K Flex instrument (Thermo Fisher Scientific; formerly Life Technologies, Grand Island, NY). This analysis was performed on the single nucleotide polymorphisms (SNPs) identified in the original cohort of 46 IBD patients that achieved a *p* value of 0.1 or less and included the polymorphisms rs4073113 (*p* = 0.009), rs4234134 (*p* = 0.057), and rs6795970 (*p* = 0.096). Twenty nanograms of genomic DNA were amplified per the manufacturer's directions and scaled to a total volume of 5 μL in an Applied Biosystems® Veriti® 384-well thermal cycler for each of the above assay IDs.

### Statistical analysis

Patients' baseline and clinical characteristics were summarized as descriptive statistics. Chi-squared or Fisher's exact test was used to compare categorical variables between groups, while independent *t*-test or one-way ANOVA was used to compare continuous variables between groups as appropriate. Fisher's exact test was used to analyze the tables of genotypic and allelic frequencies. Only one polymorphism identified from the targeted exome sequencing performed on the original 46-person IBD cohort approached a significantly higher incidence in the hypoalgesic IBD cohort (rs6795970). This variant was also the only exonic polymorphism that encoded a non-synonymous amino acid change and this SNP has previously been associated with hyposensitivity to somatosensory pain stimulation (22). As a result, we decided to focus the remainder of our analysis primarily on this variant. Data analyses were performed using GraphPad Prism v.7.0a (La Jolla, CA) or R 3.4.1 (R Core Team for Statistical Computing, Vienna, Austria, http://www.R-project.org). All *p* values of < 0.05 were considered statistically significant.

### Ethical considerations

All of the work described herein was performed following the guidelines set forth by and with the permission of the Penn State College of Medicine Institutional Review Board. All study participants gave written informed consent in accordance with the Declaration of Helsinki.

## Results

### Study participant characteristics

We identified and evaluated 121 IBD patients (41 hypoalgesic IBD, 80 other IBD) and 86 healthy controls (Table 1). The IBD cohorts had similar mean ages (and mean disease durations) while the healthy controls were significantly older (*p* < 0.0001). Each cohort had similar gender distributions. The IBD cohorts were each composed primarily of CD patients, in statistically similar proportions. There were no significant differences in disease location [using the Montreal Classification system (17)] in either the CD or UC patients when comparing the hypoalgesic and other IBD cohorts. Of note, the hypoalgesic IBD cohort was found to have significantly more strictures (*p* = 0.001), fistulae (*p* = 0.007), and abscesses (*p* = 0.003) when compared to other IBD cohort.

### Exon sequences of SCN9A, SCN10A, and SCN11A from targeted exome next-generation sequencing

As described above, DNA samples from 45 IBD patients were initially subjected to targeted exome next-generation sequencing. Overall, the mean read depth for the targeted exome sequence was 85x, with 79% of the exome covered at least 59x, and 87% of the targeted sequence covered at a read depth of 50x or more. A total of 49 exonic, single nucleotide polymorphisms (SNPs) were observed in the targeted deep sequencing of the 45 IBD patients as called using the GATK Best Practice pipeline (Table 2). SCN9A demonstrated 14 SNPs, SCN10A had 22 SNPs, and SCN11A had 13 SNPs. In order to confirm the findings of the targeted exome sequencing, three SNPs (1 in SCN10A and 2 in SCN11A, all in bold in Table 3) were analyzed by TaqMan in all 207 study participants. The results matched 100% to the targeted exome sequencing results in the original 45 patients.

**Table 2:** Single nucleotide polymorphisms (SNPs) identified for the SCN9A, SCN10A, and SCN11A genes in our initial study population.

**SCN9A**			**SCN10A**			**SCN11A**		
**SNP**	**Chrom**	**Position**	**SNP**	**Chrom**	**Position**	**SNP**	**Chrom**	**Position**
rs149207258	2	166199827	rs6790627	3	38748833	rs4234134	3	38887970
rs4303728	2	166199918	rs11711062	3	38753732	rs4234133	3	38888021
rs188336294	2	166226651	rs6771157	3	38763863	rs192005503	3	38888085
rs6746030	2	166242648	rs12632942	3	38764998	rs4640498	3	38888227
rs74401238	2	166251875	rs6795970	3	38766675	rs72869687	3	38888735
rs41268673	2	166284599	rs6791171	3	38766701	rs62244134	3	38888764
rs58022607	2	166286469	rs73062575	3	38766760	rs78953918	3	38892069
rs201531206	2	166286540	rs59468016	3	38768247	rs148945365	3	38908944
rs6747673	2	166288464	rs57326399	3	38768300	rs33985936	3	38936134
rs13402180	2	166288485	rs7374804	3	38768334	rs4073113	3	38945560
rs58465962	2	166288596	rsN/A	3	38768347	rs78812474	3	38991598
rs13414203	2	166288632	rsN/A	3	38768353	rsN/A	3	38991922
rs9646771	2	166306533	rsN/A	3	38768354	rs73068589	3	38992033
rs6432901	2	166311583	rsN/A	3	38768355		
			rsN/A	3	38768362		
			rs146028829	3	38770198		
			rs7630989	3	38793940		
			rs7617919	3	38793989		
			rs62244070	3	38798171		
			rsN/A	3	38802773		
			rs74717885	3	38805069		
			rs34314583	3	38835457		

**Table 3:** Relative frequency of SCN9A, SCN10A, and SCN11A SNPs in “Hypoalgesic IBD” compared to other IBD patients.

**ID**	**Gene**	**Residue**	**Major**	**Minor**	**“Hypoalgesic IBD”**	**“Other IBD”**	**Odds**	***P*-value**
		**change**	**Allele**	**Allele**	**risk allele Freq**.	**risk allele Freq**.	**Ratio**	
*rs4073113*	*SCN11A*	C546C*	*A*	*G*	*84.8*	*59.1*	*3.8571*	*0.0093*
rs4234134	SCN11A	3' UTR	T	A	39.1	61.4	0.4048	0.0571
*rs6795970*	*SCN10A*	V1073A*	*A*	*G*	*54.3*	*36.4*	*2.0833*	*0.0958*
rs73062575	SCN10A	P1044T	G	T	0.0	6.8	0.1275	0.1127
rs4234133	SCN11A	3' UTR	T	C	39.1	56.8	0.4886	0.1391
rs6791171	SCN10A	T1063T	C	T	10.9	2.3	5.2439	0.2035
rs41268673	SCN9A	P610T	G	T	0.0	4.5	0.1828	0.2362
rs41268673	SCN9A	P610T	G	T	0.0	4.5	0.1828	0.2362
rs78953918	SCN11A	T1410T	C	T	0.0	4.5	0.1828	0.2362
rs74717885	SCN10A	I206M	T	C	6.5	0.0	7.1609	0.2419
rs: N/A	SCN11A	3'UTR	C	T	0.0	0.0	0.4889	0.3118
rs62244134	SCN11A	Y1599Y	G	A	15.2	6.8	2.4530	0.3161
rs7630989	SCN10A	S509P	A	G	8.7	2.3	4.0952	0.3614
rs6746030	SCN9A	R1150W	A	G	80.4	88.6	0.5271	0.3856
rs33985936	SCN11A	V909I	C	T	19.6	27.3	0.6486	0.4589
rs7374804	SCN10A	K950K	T	C	13.0	6.8	2.0500	0.4856
rs11711062	SCN10A	S1336T	A	T	0.0	2.3	0.3118	0.4889
rs: N/A	SCN10A	L265V	G	C	0.0	2.3	0.3118	0.4889
rs13414203	SCN9A	A373A	A	G	28.3	36.4	0.6894	0.5002
rs9646771	SCN9A	P148P	T	C	56.5	63.6	0.7429	0.5250
rs73068589	SCN11A	5' UTR	C	A	10.9	15.9	0.6446	0.5464
rs6790627	SCN10A	K1440K	T	C	13.0	18.2	0.6750	0.5693
rs59468016	SCN10A	G979G	G	A	21.7	15.9	1.4683	0.5931
rs13402180	SCN9A	E422E	T	C	28.3	34.1	0.7616	0.6503
rs6747673	SCN9A	R429R	A	T	39.1	45.5	0.7714	0.6699
rs149207258	SCN9A	V1593V	C	A	4.3	6.8	0.6212	0.6733
rs78812474	SCN11A	R86R	G	T	4.3	6.8	0.6212	0.6733
rs7617919	SCN10A	L492L	G	A	17.4	13.6	1.3333	0.7731
rs6771157	SCN10A	T1130T	G	C	19.6	15.9	1.2857	0.7846
rs12632942	SCN10A	L1091P	A	G	19.6	15.9	1.2857	0.7846
rs57326399	SCN10A	I962V	T	C	21.7	18.2	1.2500	0.7939
rs6432901	SCN9A	Q58Q	C	T	56.5	59.1	0.9000	0.8338
rs62244070	SCN10A	E428E	C	T	17.4	15.9	1.1128	1.0000
rs188336294	SCN9A	V1427V	G	A	2.2	0.0	2.9341	1.0000
rs74401238	SCN9A	R1110Q	C	T	2.2	0.0	2.9341	1.0000
rs58022607	SCN9A	S490N	C	T	2.2	0.0	2.9341	1.0000
rs201531206	SCN9A	S466S	G	A	2.2	0.0	2.9341	1.0000
rs58465962	SCN9A	V385V	C	A	2.2	0.0	2.9341	1.0000
rs: N/A	SCN10A	E848G	T	C	2.2	2.3	0.9556	1.0000
rs: N/A	SCN10A	E846Q	T	A	2.2	2.3	0.9556	1.0000
rs: N/A	SCN10A	E846V	C	G	2.2	2.3	0.9556	1.0000

*Asterisks denote SNPs that were confirmed by Taqman PCR genotyping. Of note, the deep sequencing and PCR genotyping were 100% concordant*.

### SNP association analysis: comparing “hypoalgesic IBD” patients with other IBD patients

Table 3 presents the odds ratios for the 49 SNPs observed in the deep sequencing of all the IBD patients (arranged from most statistically significant to least). Three SNPs [rs4073113 (*p* = 0.009), rs4234134 (*p* = 0.057), and rs6795970 (*p* = 0.096)] demonstrated differential expression in “hypoalgesic IBD” patients when compared to all other IBD patients exhibiting a *p* value of 0.1 or less (Fisher's exact test).

The rs6795970 polymorphism produces a non-synonymous amino acid change (alanine at amino acid 1073 to valine in *SCN10A*; A1073V). This functional polymorphism was therefore examined in all 121 IBD patients. Homozygotic frequency for the rs6795970 polymorphism was significantly higher in the hypoalgesic IBD cohort when compared to the other IBD patients (*p* = 0.01) (Table 4). This was not the case when hypoalgesic IBD patients were compared to healthy controls (Table 5). Of note, no differences in homozygotic frequency were found for the other SNP described above (rs4073113) or the other SNPs outlined in Tables 2, 3 when comparing the three study cohorts (hypoalgesic IBD, other IBD or controls).

**Table 4:** Genotypic and allelic frequencies of the SCN10A polymorphism, rs6795970 in Hypoalgesic IBD vs. “Other IBD” patients.

	**“Hypoalgesic IBD”**	**Other IBD**	***p*-value**
**Genotypic frequency (SCN10A, Na**_v_**1.8)**	**(*****n*** = **41)**	**(*****n*** = **80)**
A/A (1073Val/Val)	19 (46%)	18 (22%)	0.01
A/G (1073Val/Ala)	14 (34%)	45 (55%)
G/G (1073Ala/Ala)	8 (20%)	17 (23%)
**Allelic frequency**	**(*****n*** = **82)**	**(*****n*** = **160)**
A (valine)	52 (63%)	91 (57%)	0.34
G (alanine)	30 (37%)	69 (43%)

*Frequencies are listed as number of subjects (% of genotypic or allelic total). The homozygous genotype (A/A) for the rs6795970 polymorphism was significantly more common in the Hypoalgesic IBD group (p = 0.01, Fisher's Exact Test). The allelic frequency of this polymorphism was not more common in the Hypoalgesic IBD group (p = 0.34, Fisher's Exact Test). G, wild type; A, rs6795970 polymorphism (c.3218G > A). Notably, according to the 1,000 Genomes sequencing project, the A allele is present at a frequency of 29% of the Caucasian population (the primary demographic of this study group)*.

**Table 5:** Genotypic and allelic frequencies of the SCN10A polymorphism, rs6795970 in Hypoalgesic IBD vs. Healthy Control patients.

	**“Hypoalgesic IBD”**	**Healthy Controls**	***p*-value**
**Genotypic frequency (SCN10A, Na**_v_**1.8)**	**(*****n*** = **41)**	**(*****n*** = **86)**
A/A (1073^Val/Val^)	19 (46%)	30 (35%)	0.25
A/G (1073^Val/Ala^)	14 (34%)	40 (47%)
G/G (1073^Ala/Ala^)	8 (20%)	16 (18%)
**Allelic frequency**	**(*****n*** = **82)**	**(*****n*** = **172)**
A (valine)	52 (63%)	100 (58%)	0.49
G (alanine)	30 (37%)	72 (42%)

*Frequencies are listed as number of subjects (% of genotypic or allelic total). The homozygous genotype (A/A) for the rs6795970 polymorphism was not significantly different between the cohorts (p = 0.25, Fisher's Exact Test). Allelic frequency of this polymorphism was also not significantly different (p = 0.49, Fisher's Exact Test). G, wild type; A, rs6795970 polymorphism (c.3218G > A). Of note, according to the 1,000 Genomes sequencing project, The “A” allele is present at a frequency of 29% of the Caucasian population (the primary demographic of this study group)*.

## Discussion

This investigation of three VGSC genes, SCN9A, SCN10A, and SCN11A, demonstrated that “hypoalgesic IBD” patients had a statistically higher prevalence of homozygosity for one SNP: rs6795970 (SCN10A). This polymorphism encodes a non-synonymous amino acid substitution (alanine at amino acid 1073 to valine in *SCN10A*; A1073V). As indicated above, this SNP demonstrated an increased homozygotic frequency in the “hypoalgesic IBD” cohort compared to other IBD patients. This finding is significant for a number of reasons. Although other studies have provided evidence for links between specific SNPs and syndromes associated with visceral hypersensitivity (including irritable bowel syndrome [Na_V_1.5, (23)] and functional dyspepsia [Na_V_1.8, (24)], this is the first demonstration of an association between an abdominal pain phenotype in IBD and a particular genetic variant. This is also one of the first SNPs associated with diminished pain experience in a gastrointestinal disorder. It is important to note that the significant association described above is specifically with homozygosity for rs6795970 (that is, for individuals that presumably only have the polymorphic channel). Heterozygotes do not display a variant frequency, suggesting that one “wild-type” copy is sufficient to protect individuals from the associated physiological alterations that result in hypoalgesic and/or “silent” IBD.

There is growing evidence that this particular polymorphism (rs6795970) and the associated Na_V_1.8 channel have significant influence on visceral pain perception. The *SCN10A* gene encodes the alpha subunit of Na_V_1.8 (22, 25, 26). This channel is predominantly expressed in the periphery and, as previously indicated, appears to play a critical role in pain transmission. A functional assessment of this polymorphism by Duan and colleagues revealed that this same genetic variant (rs6795970) results in altered electrophysiological function of the Nav1.8 channel and higher thresholds for mechanical pain in a discovery cohort (22). Functional assessments have shown that the minor allele of rs6795970 (valine-1073) shifts sodium channel activation, resulting in reduced repetitive firing of dorsal root ganglion neurons, thereby lowering mechanical pain sensitivity (23). This is the first study in humans to demonstrate a link between a genetic variant of Na_V_1.8 and intestinal pain perception in the setting of IBD (or any other gastrointestinal disorder). This is an important finding that provides key insights regarding visceral nociceptive physiology and new potential diagnostic and therapeutic targets to consider in patients with disorders of abdominal pain perception.

We are confident in the results of this investigation for several reasons. Our study determined current IBD disease activity through direct endoscopic evaluation of the intestinal mucosa and coupled this information with contemporary validated survey results relating to patient abdominal pain experience. We performed a comprehensive evaluation and analysis of the three VGSC genes of interest (SCN9A, SCN10A, and SCN11A) and compared all of the resultant SNPs (totaling 49 independent polymorphisms) among the study cohorts. The initial genetic findings were “double-checked” by Taqman PCR and additional samples were evaluated using the same methodology. These results make us feel more assured of the findings and the significant influence that the rs6795970 SNP has in this context, particularly because it was the only variant encoding a non-synonymous amino acid change that was found to be significantly different in prevalence among any of the study cohorts.

Potential limitations of this study include the relatively small sample size and the fact that it was performed using individuals from a single medical center. It will be important to independently validate the results of this investigation with larger numbers of the same patient cohorts. It would also be informative to perform this analysis in other conditions associated with chronic abdominal pain, including irritable bowel syndrome. Our determination of hypoalgesic IBD was also based upon the survey responses and colonoscopic findings from one clinical encounter. It would be useful to determine how consistent these findings (and the study cohorts) were between different time points. Additionally, our cohort of interest focused only on abdominal pain experience and did not include consideration of other symptoms frequently associated with IBD, such as diarrhea, bleeding, and bloating. We utilized this approach in order to minimize potential confounding that could occur in the process of interpreting our data. However, future analyses incorporating individuals lacking the additional symptoms described above could provide a more complete understanding of the so-called “silent” IBD phenotype, which has been associated with lack of perception and/or reporting of any symptom that could be associated with this disorder. The mean allelic frequency of the rs6795970 SNP in our total cohort was ~59%, which is significantly higher than that previously reported [e.g., 1000 Genomes Project MAF = 29% (27, 28)] suggesting a potential founder's effect in our population (though this should not impact the relative differences in allelic or genotypic frequency found in this study). While we focused on the non-synonymous polymorphism, rs6795970, it is possible that the synonymous and untranslated region variants described above could also play a role in visceral pain perception, for example through differential codon usage or alteration of mRNA levels. Additionally, we used a mixed population of CD and UC patients. These conditions have varied potential mechanisms for inducing chronic abdominal pain that may not necessarily overlap neatly with one another and there is certainly reason to investigate larger, adequately powered cohorts of both CD and UC in this context. Finally, although validated surveys were utilized to provide information about patient abdominal pain experience, there was no objective measure of visceral nociceptive perception.

Despite these drawbacks, we believe the results of this study provide compelling evidence that Na_V_1.8 is important to visceral nociceptive function, particularly in the setting of IBD. In addition to confirming this concept in a larger population of IBD patients, it will also be important to evaluate healthy control cohorts in addition to other gastrointestinal conditions associated with alterations in visceral sensation (e.g., irritable bowel syndrome) to determine whether the impact of this channel and the genetic variant described above is limited to inflammatory conditions of the gut. It will also be important to further evaluate the relationship between the rs6795970 polymorphism, and Na_V_1.8 function in general, and visceral pain perception through mechanistic studies incorporating targeted genetic changes and objective measures of pain response (e.g., visceromotor response in animal knock-in models).

If the studies described above further substantiate the link between Na_V_1.8 and pain perception in IBD (and potentially other gut disorders associated with chronic abdominal pain), this will represent a novel, new target to help identify patients at higher risk of major disease-associated complications. It could also serve as a new model to help develop less toxic pain therapies in patients with chronic abdominal pain. This could help to minimize the use of other frequently used medication types associated with increased morbidity (e.g., NSAID's, opiates) while also more intelligently targeting the sensory systems associated with the pain, thereby reducing the use of high cost healthcare resources (e.g., emergency room visits, imaging, surgery) in the name of evaluating or treating chronic abdominal pain.

## Author contributions

EG-L helped to develop some of the conceptual framework for this study, performed the TaqMan sequencing experiments, and undertook some of the genetic analyses and was a key author and editor of this manuscript. YI helped develop the probes used for targeted exome sequencing, provided regular conceptual consultation, and helped to write and edit the manuscript. VW and LZ helped to select and perform the statistical tests and reviewed the manuscript. WK and XH provided the blood samples and patient data evaluated in this study and both reviewed the manuscript. KV and MC developed the major framework for this study, performed the primary analyses associated with this investigation, and were key, equal authors, and editors of this manuscript.

### Conflict of interest statement

The authors declare that the research was conducted in the absence of any commercial or financial relationships that could be construed as a potential conflict of interest.

## References

[1] Dahlhamer JM Zammitti EP Ward BW Wheaton AG Croft JB. Prevalence of inflammatory bowel disease among adults aged ≥18 Years - United States, 2015. MMWR Morb Mortal Wkly Rep. (2016) 65:1166–9. 10.15585/mmwr.mm6542a3 27787492

[2] Norton C Czuber-Dochan W Artom M Sweeney L Hart A. Systematic review: interventions for abdominal pain management in inflammatory bowel disease. Aliment Pharmacol Ther. (2017) 46:115–25. 10.1111/apt.14108 28470846

[3] Bielefeldt K Davis B Binion DG. Pain and inflammatory bowel disease. Inflamm Bowel Dis. (2009) 15:778–88. 10.1002/ibd.20848 19130619 PMC3180862

[4] af Bjorkesten CG Nieminen U Turunen U Arkkila P Sipponen T Farkkila M. Surrogate markers and clinical indices, alone or combined, as indicators for endoscopic remission in anti-TNF-treated luminal Crohn's disease. Scand J Gastroenterol. (2012) 47:528–37. 10.3109/00365521.2012.660542 22356594

[5] Peyrin-Biroulet L Reinisch W Colombel JF Mantzaris GJ Kornbluth A Diamond R. Clinical disease activity, C-reactive protein normalisation and mucosal healing in Crohn's disease in the SONIC trial. Gut (2014) 63:88–95. 10.1136/gutjnl-2013-304984 23974954

[6] Click B Vargas EJ Anderson AM Proksell S Koutroubakis IE Ramos Rivers C. Silent Crohn's disease: asymptomatic patients with elevated C-reactive protein are at risk for subsequent hospitalization. Inflamm Bowel Dis. (2015) 21:2254–61. 10.1097/MIB.0000000000000516 26197446

[7] Coates MD Vrana KE Ruiz-Velasco V The influence of voltage-gated sodium channels on human gastrointestinal nociception. Neurogastroenterol Motil (2018) 2018:e13460 10.1111/nmo.13460 30216585

[8] Bielefeldt K Ozaki N Gebhart GF. Mild gastritis alters voltage-sensitive sodium currents in gastric sensory neurons in rats. Gastroenterology (2002) 122:752–61. 10.1053/gast.2002.31901 11875008

[9] Bielefeldt K Ozaki N Gebhart GF. Experimental ulcers alter voltage-sensitive sodium currents in rat gastric sensory neurons. Gastroenterology (2002) 122:394–405. 10.1053/gast.2002.31026 11832454

[10] Moore BA Stewart TM Hill C Vanner SJ. TNBS ileitis evokes hyperexcitability and changes in ionic membrane properties of nociceptive DRG neurons. Am J Physiol Gastrointest Liver Physiol. (2002) 282:G1045–51. 10.1152/ajpgi.00406.2001 12016130

[11] Su X Wachtel RE Gebhart GF. Capsaicin sensitivity and voltage-gated sodium currents in colon sensory neurons from rat dorsal root ganglia. Am J Physiol. (1999) 277:G1180–8. 10.1152/ajpgi.1999.277.6.G1180 10600815

[12] Beyak MJ Ramji N Krol KM Kawaja MD Vanner SJ. Two TTX-resistant Na+ currents in mouse colonic dorsal root ganglia neurons and their role in colitis-induced hyperexcitability. Am J Physiol Gastrointest Liver Physiol. (2004) 287:G845–55. 10.1152/ajpgi.00154.2004 15205116

[13] Akopian AN Sivilotti L Wood JN. A tetrodotoxin-resistant voltage-gated sodium channel expressed by sensory neurons. Nature (1996) 379:257–62. 10.1038/379257a0 8538791

[14] Gold MS Zhang L Wrigley DL Traub RJ. Prostaglandin E(2) modulates TTX-R I(Na) in rat colonic sensory neurons. J Neurophysiol. (2002) 88:1512–22. 10.1152/jn.2002.88.3.1512 12205171

[15] Jarvis MF Honore P Shieh CC Chapman M Joshi S Zhang XF. A-803467, a potent and selective Nav1.8 sodium channel blocker, attenuates neuropathic and inflammatory pain in the rat. Proc Natl Acad Sci USA. (2007) 104:8520–5. 10.1073/pnas.0611364104 17483457 PMC1895982

[16] Laird JM Souslova V Wood JN Cervero F. Deficits in visceral pain and referred hyperalgesia in Nav1.8 (SNS/PN3)-null mice. J Neurosci. (2002) 22:8352–6. 10.1523/JNEUROSCI.22-19-08352.2002 12351708 PMC6757795

[17] Satsangi J Silverberg MS Vermeire S Colombel JF. The Montreal classification of inflammatory bowel disease: controversies, consensus, and implications. Gut (2006) 55:749–53. 10.1136/gut.2005.082909 16698746 PMC1856208

[18] Peyrin-Biroulet L Panes J Sandborn WJ Vermeire S Danese S Feagan BG. Defining disease severity in inflammatory bowel diseases: current and future directions. Clin Gastroenterol Hepatol. (2016) 14:348–54.e317. 10.1016/j.cgh.2015.06.001 26071941

[19] Irvine EJ Zhou Q Thompson AK. The short inflammatory bowel disease questionnaire: a quality of life instrument for community physicians managing inflammatory bowel disease. CCRPT Investigators. Canadian Crohn's Relapse Prevention Trial. Am J Gastroenterol. (1996) 91:1571–8. 8759664

[20] Li H Durbin R. Fast and accurate short read alignment with Burrows-Wheeler transform. Bioinformatics (2009) 25:1754–60. 10.1093/bioinformatics/btp324 19451168 PMC2705234

[21] Wang K Li M Hakonarson H. ANNOVAR: functional annotation of genetic variants from high-throughput sequencing data. Nucleic Acids Res. (2010) 38:e164. 10.1093/nar/gkq603 20601685 PMC2938201

[22] Duan G Han C Wang Q Guo S Zhang Y Ying Y. A SCN10A SNP biases human pain sensitivity. Mol Pain (2016) 12:1744806916666083. 10.1177/1744806916666083 27590072 PMC5011395

[23] Saito YA Strege PR Tester DJ Locke GR 3rd Talley NJ Bernard CE. Sodium channel mutation in irritable bowel syndrome: evidence for an ion channelopathy. Am J Physiol Gastrointest Liver Physiol. (2009) 296:G211–8. 10.1152/ajpgi.90571.2008 19056759 PMC2643921

[24] Arisawa T Tahara T Shiroeda H Minato T Matsue Y Saito T. Genetic polymorphisms of SCN10A are associated with functional dyspepsia in Japanese subjects. J Gastroenterol. (2013) 48:73–80. 10.1007/s00535-012-0602-3 22618805

[25] Abou Ziki MD Seidelmann SB Smith E Atteya G Jiang Y Fernandes RG Deleterious protein-altering mutations in the SCN10A voltage-gated sodium channel gene are associated with prolonged QT. Clin Genet. (2017) 93:741–51. 10.1111/cge.13036 28407228 PMC5640462

[26] Savio-Galimberti E Weeke P Muhammad R Blair M Ansari S Short L. SCN10A/Nav1.8 modulation of peak and late sodium currents in patients with early onset atrial fibrillation. Cardiovasc Res. (2014) 104:355–63. 10.1093/cvr/cvu170 25053638 PMC4271018

[27] Chambers JC Zhao J Terracciano CM Bezzina CR Zhang W Kaba R. Genetic variation in SCN10A influences cardiac conduction. Nat Genet. (2010) 42:149–52. 10.1038/ng.516 20062061

[28] Delaney JT Muhammad R Shi Y Schildcrout JS Blair M Short L. Common SCN10A variants modulate PR interval and heart rate response during atrial fibrillation. Europace (2014) 16:485–90. 10.1093/europace/eut278 24072447 PMC3977588

